# Reflections on Co-production as a Mode of Knowledge Production: Comment on "Research Coproduction: An Underused Pathway to Impact"

**DOI:** 10.34172/ijhpm.8909

**Published:** 2025-03-09

**Authors:** Carole A. Estabrooks

**Affiliations:** Faculty of Nursing, University of Alberta, Edmonton, AB, Canada.

**Keywords:** Co-Production, Implementation Science, Integrated Knowledge Translation, Systems and Policy

## Abstract

Rycroft-Malone and colleagues’ editorial on research co-production highlights the potential of a co-production mode of research to narrow the gap between knowledge production and use. This commentary critiques implicit assumptions within the argument and challenges the view that traditional (Mode 1) science bears the primary responsibility for delayed implementation and questions the inherent superiority of co-production. It also highlights the importance of political and policy considerations in considering research uptake. "Mode 3" knowledge production (integrating Modes 1 and 2 discovery) offers a potentially more advanced framework that recognizes systems and organizational perspectives. A deeper, multi-layered exploration of the influence of socio-political and policy contexts is needed to understand the full potential of co-production on knowledge utilization.

 In 2024, Rycroft-Malone and colleagues published a thoughtful and sophisticated editorial, *Research Coproduction: An Underused Pathway to Impact.*^1^ They argued that the actual use of research and its derivatives (eg, evidence-informed guidelines, recommendations, etc) is a function of “how we create the evidence” and suggest that “how we create the evidence” influences our ability to narrow the long-standing gap from knowledge production to knowledge use. They further argue that the solution to these important issues lies in the adoption of a “co-production” mode of research or discovery. They go on to describe well-known challenges in co-production at micro to macro, meso, and micro levels. Here I offer comments about their editorial, suggesting some areas of deeper exploration. As with all things that we write, their editorial carries with it *implicit *assumptions and I try to point out the ones that trouble me the most, most particularly that traditional science carries most or all of the responsibility for failures to get research evidence to use more quickly and that co-production may be a superior form of discovery.

 At the macro level they address the broadening societal orientation (at least in some societies) towards the democratization of knowledge, and toward equity, diversity, inclusion and social justice. This bears directly on who is included among the co producers of knowledge and our understandings of “the calculus,” by which we determine whose knowledge matters or in our complex geo-political worlds who “wins.” We see dramatic examples in some western (still) democracies of who wins and it is often not science. For example, the science on climate change is clear if evolving – for every degree of warming predictable and catastrophic changes can be expected. Yet we see major pressures on these provisional truths of science, often driven by greed (the fossil fuel industry), fear (any industry where people fear the loss of their jobs), political goals, and many other motivations. These sometimes stunningly robust efforts to repel science are driven by powerful disinformation campaigns and sophisticated use of social media. They are geopolitical in nature. In the face of this, science can seem ill equipped to advance. Thus, exposing one area the authors did not touch upon, the broader and always complex political *and* policy arenas. I submit that without a common-sense appreciation of the political arena and a robust understanding of at least the policy arena, that who gets on research teams and how knowledge is produced actually matters very little sometimes. Implementation science has emerged in the last 30 years relatively separate from the long history of policy studies and analyses, creating a noticeable gap in our appreciation of how knowledge actually get used or not. There are of course many examples at macro levels of collaborations and partnerships, the World Health Organization (WHO) driven one with respect to rehabilitation is a strong example.^2^ However, generally in the research arena implementation science has not been as closely aligned with policy studies as it could be.

 At the meso level, the authors outline some of the most commonly cited challenges in advancing a co-production model. They outline familiar and still unresolved challenges, in particular at the levels of academic institutions and research funders. An implicit belief that I suspect underlies the views of all authors writing about these challenges is that we (universities, research funders) continue to privilege what Gibbons et al^3^ call Mode 1 (traditional) science. We know of course that many of the discoveries of all science including Mode 1 science lag in implementation let alone spread and scaling. However, if this privileging of traditional scientific production is to blame for the universities and funders not making the significant changes that need to be done, then it might be useful to probe case studies of first why many Mode 1 discoveries also lagged on the “needs putting to use” measure. Take the discovery of *Helicobacter pylori* as a cause of gastric ulcers in 1982^4^ and the extensive lag until practice changed and patients were prescribed an antibiotic. If we contrasted the many examples of lags of Mode 1 discovery against some of the more startling discoveries that were rapidly and broadly adopted (eg, penicillin, polio and COVID-19 vaccination) we might uncover a deeper understanding that included a realization that all blame for discoveries not being adopted rapidly may not rest with traditional science. In fact, at least since the halcyon days of very early vaccine discoveries with profound public health impact and since the advent of large multi-national pharma and the advents of powerful social media and other advertising, the speed of “bench to citizen” has become intertwined with and influenced by multiple factors that have much to do with complex and powerful partnerships in the industrial-academic complex that extend beyond the intention in Rycroft-Malone and colleagues’ editorial and this commentary. Co-production is not a panacea for narrowing the gap; it does however, have incredibly important applications. It is true that that research funders and universities have been delinquent in paying more than lip service to the complex and unique needs of this form of science. Still after all this time. However, it is just too simple to imply that Mode 1 knowledge production (traditional science) is not closing the gap or has not been engaged in these monetized partnerships that may or may not be co-production in the sense of this commentary, but that a “Mode 2 like” approach will.^3,5^

 Mode 2 knowledge production^3,5^ involves nonhierarchical relationships with stakeholders to collaborate on a research issue situated in a specific health care context. In this sense, it is based on the needs of end users and is arguably a more socially accountable form of knowledge production. This is in contrast to the more traditional form of knowledge production, Mode I knowledge production which reflects the traditional, academic norms of scholarship in the disciplines and institutions in which researchers work, such as academic tenure and promotion based on high impact, peer-reviewed publication.^3,5^ Its foundations rest on principles of scientific expertise, peer review, and non-interference.^6^ More recently Carayannis and colleagues^7,8^ describe Mode 3 knowledge production, loosely an attempt to bridge Modes 1 and 2 discovery. As I and my team have evolved over the last 20 years in trying to practice what we call *integrated knowledge translation* and what Rycroft and colleagues describe as co-production, I have become increasingly convinced we need to work toward this Mode 3 form of discovery. It is a challenging space in which to work and perhaps an even more radical agenda than Gibbons’ Mode 2. It requires a robust understanding of systems thinking, of organizational behaviours, and an openness to continuously broadening the constituents that are engaged in the program of research – commonly not taught in professional disciples who make up the majority of health researchers. It requires an almost inexhaustible armamentarium of knowledge, skill and experience in multiple areas – and especially, as Rycroft Malone et al point out, in relationship building and maintenance, organization and systems knowledge and thinking. One must integrate a wide array of research partners, such as researchers who are genuinely interested in and willing to engage in systems and organization thinking, and partners with some basic and realistic understanding of policy formation and implementation. It also requires that one is willing and able to do programmatic research and to conduct this kind of research in a near vacuum of support from many universities and almost all research funders. These challenges at the micro level are not treated in depth by Rycroft Malone and colleagues who focus more on the macro and meso areas. Examples of co-production at the micro level offer potent opportunities for change at the interface of patients and care givers and the systems in which they interact. For example, working with often neglected direct care workers and their leaders in long term care (or nursing home) settings is a rich area of science. Not doing so has resulted in many failed improvement and implementation initiatives. To implement for example, a trauma informed approach to care in long term care without working closely with the occupational group that makes up about 90% of direct care provision seems doomed to fail. But historically, quality efforts have come from the top down and when they have been tried from the bottom up, have not appreciated the important interactions and synergies of partners at multiple levels in a complex adaptive micro system like a nursing home. To address the *knowledge production – knowledge uptake gap* at the micro level requires serious attention to not only the meso level and the forces at the macro level, but also to a fundamentally more sophisticated approach to the teaching and support of young researchers that is currently almost nonexistent in the teachings of our higher educational settings.

## Conclusion

 Rycroft Malone and colleagues published a succinct and important editorial. Their editorial offers a sharp reminder that co-production does not function in a vacuum or on the fringes or in some “separate department,” but rather is deeply embedded in multiple system layers and in a constantly changing societal context. Their editorial offers welcome evidence of a rapidly maturing understanding in this area of research and suggests that this team may be at the forefront of these advancements.

## Ethical issues

 Not applicable.

## Conflicts of interest

 Author declares that she has no conflicts of interest.
